# Automation of surgical skill assessment using a three-stage machine learning algorithm

**DOI:** 10.1038/s41598-021-84295-6

**Published:** 2021-03-04

**Authors:** Joël L. Lavanchy, Joel Zindel, Kadir Kirtac, Isabell Twick, Enes Hosgor, Daniel Candinas, Guido Beldi

**Affiliations:** 1 Department of Visceral Surgery and Medicine, Inselspital, Bern University Hospital, University of Bern, 3010 Bern, Switzerland; 2 Caresyntax, Komturstr. 18A, 12099 Berlin, Germany

**Keywords:** Translational research, Biomedical engineering

## Abstract

Surgical skills are associated with clinical outcomes. To improve surgical skills and thereby reduce adverse outcomes, continuous surgical training and feedback is required. Currently, assessment of surgical skills is a manual and time-consuming process which is prone to subjective interpretation. This study aims to automate surgical skill assessment in laparoscopic cholecystectomy videos using machine learning algorithms. To address this, a three-stage machine learning method is proposed: first, a Convolutional Neural Network was trained to identify and localize surgical instruments. Second, motion features were extracted from the detected instrument localizations throughout time. Third, a linear regression model was trained based on the extracted motion features to predict surgical skills. This three-stage modeling approach achieved an accuracy of 87 ± 0.2% in distinguishing good versus poor surgical skill. While the technique cannot reliably quantify the degree of surgical skill yet it represents an important advance towards automation of surgical skill assessment.

## Introduction

Intraoperative and postoperative complications remain a clinical challenge in surgical practice. Not only patient and procedure related factors increase the risk of adverse surgical outcomes so do poor technical skills of surgeons^1,2^. A recent study suggests that the disparity in surgical skill among practicing surgeons accounts for more than 25% of the variation in patient outcomes^3^. To improve patient outcomes, it is therefore necessary to train surgeons’ technical performance by continuously providing objective feedback on their surgical skills.

Assessing surgical skills objectively remains a matter of debate^4^. Traditionally, the skills of surgical trainees have been assessed using in vitro model trainers^5,6^. However, these approaches have been criticized for lacking reality and do not translate into reduced mortality or morbidity^7^. Common practice in vivo skill assessment is either based on direct observation of surgical trainees^8,9^ or on retrospective analysis of operation videos^10,11^. Skills of surgical trainees are rated by experts according to predefined criteria^8,12^. While these approaches are a much better reflection of reality and can be blinded, they are limited by reproducibility and rater availability^13^.

With recent advances in machine learning, the attention has shifted to automated surgical skill assessment, particularly in robotic interventions. Robotic surgeries have the advantage that kinematic data of instruments and video recordings are readily available from the console^14–18^. Most of the previous studies have solely focused on robotic kinematics data to compute automated performance metrics or predict skill levels^14–17,19,20^. One study has combined motion features extracted from video and kinematic signals^18^. Another one exclusively relied on surgical videos and utilized a 3D convolutional neural network (CNN) to capture both spatial and temporal information for surgical skill prediction^21^. Methodologies have ranged from hidden markov chains^20^ and traditional machine learning classifiers^14^, over time series feature extraction^17,18^ to CNNs^15,16,21,22^. Although these works provide an important contribution to the field their applicability in real-world clinical setting are limited as robotic surgeries are still rare and kinematics data therefore frequently not available.

To apply automated surgical skill assessment to surgical practice it is necessary that machine learning models are based on data commonly recorded in surgery such as laparoscopic videos. Numerous studies have shown that CNNs can be successfully applied to real-world laparoscopic videos^23^. Examples include procedural phase and instrument presence detection^24^ as well as surgical instrument segmentation^25^. So far only one previous study analyzed surgical skill based on laparoscopic videos^26^. Jin et al*.* used a region-based CNN to localize and identify seven surgical instruments in videos of laparoscopic cholecystectomies. They performed a descriptive analysis of five videos showing differences in instrument utilization times, instrument path length and instrument movement ranges between varying surgical skill levels. While being based on a small dataset these findings were promising and inspired us to suggest an extended modeling approach for surgical skill assessment.

Continuing the work of previous studies, we aimed to automatically assess surgical skill using laparoscopic cholecystectomy videos. As performed by Jin et al*.* we extracted instrument locations from laparoscopic videos. We then computed motion features from the instrument trajectories throughout time with the aim to capture a surgeon’s instrument handling skills. Finally, the calculated motion features were fed into a machine learning model to predict surgical skill. To simplify the problem, we focused on video segments of clip application at the end of the hepatocystic dissection, a surgical gesture that requires careful handling of the clip applier and thus displays a good proxy to rate surgical skill.

**Figure 1:**
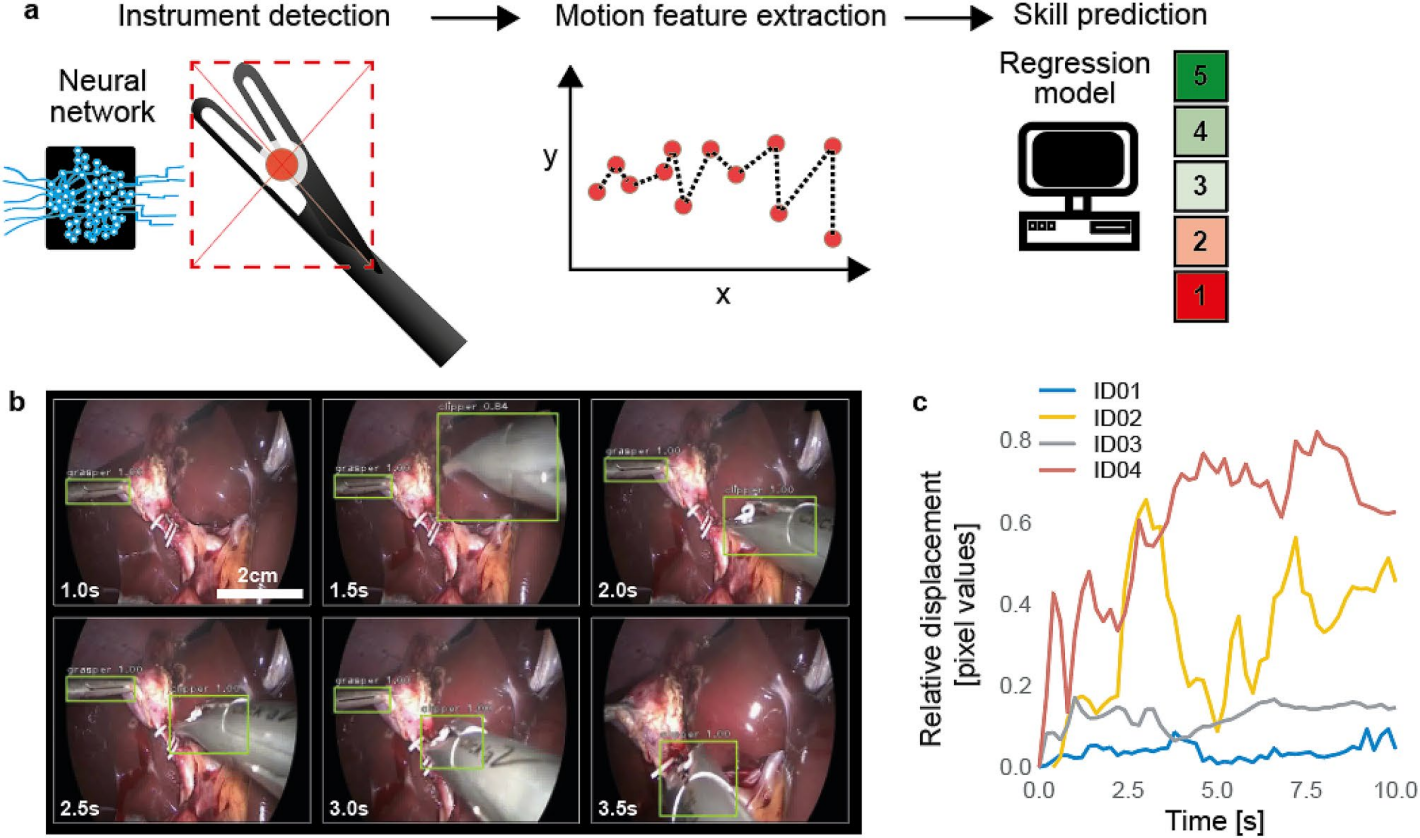
(**a**) Schematic presentation of the three-staged machine learning algorithm. First, instruments were automatically detected by a CNN in the laparoscopic videos and second, motion features were extracted. Finally the extracted motion features were used to automatically predict surgical skill using a linear regression model. (**b**) Screenshots of instrument detection algorithm (full video in the Supplementary Material Video S1). Green bounding boxes with corresponding class labels (grasper and clipper) and detection confidence. (**c**) Four random examples of relative displacement of the clipper as tracked by the instrument detection algorithm, ID01 and ID03 show a narrow range of movement, whereas ID02 and ID04 show a wide range of movement.

In the following we will describe our proposed modeling approach (Fig. 1a) in three stages: In the first stage, a Convolutional Neural Network (CNN) based classifier was trained to both identify and localize instruments in video frames. In the second stage, the instrument location predictions were transformed to time-series motion features. Finally, in the third stage, a linear regression model was trained utilizing the extracted motion features as input to predict surgical skill.

## Methods

### Ethical approval

The institutional review board—the ethics committee of the Canton of Bern—approved the study design, the use of laparoscopic videos, and waived the need to obtain informed consent (KEK 2018-01964). All methods were performed in accordance with the relevant guidelines and regulations.

### Dataset

#### Video storage and annotation

The institutional video archive was screened for video recordings of laparoscopic cholecystectomies performed between January 2014 and May 2019. A total of 242 videos were identified. The videos were stored in Movie Pictures Experts Group (MPEG) format on a secured internet-based platform (https://ala.surgery) for further processing. The videos were segmented into procedural phases of the intervention. The dissection of the hepatocystic triangle was labeled beginning with the first use of a dissection instrument in the region of the hepatocystic triangle until the cystic duct and artery were cut. Within the dissection of the heptatocystic triangle applications of surgical clips (B. Braun Aesculap Challenger Ti, Tuttlingen, Germany and Teleflex Hem-o-lok, Belp, Switzerland) were annotated. In total, 949 segments of clip applications were labeled.

#### Skill rating

Surgical skills can be assessed globally per intervention or specifically on the level of procedural phases or surgical gestures. In this study clip application at the end of the hepatocystic dissection phase served as the surgical gesture used as a proxy for surgical skill. A total of 949 clip applications in 242 video recordings of laparoscopic cholecystectomy were rated by four board certified surgeons (Table 1). Skill ratings were based on a Likert scale from 1 (minimum score) to 5 (maximum score) (definitions see Supplementary Table S1).

**Table 1:** Dataset statistics.

	Min	Max	Mean	Std dev	Sum
# clips per video	1	9	3.92	1.75	949
Clip duration (s)	1	89	15.13	9.91	14,361
Average rating	1	5	3.7	1.02	3514

The distribution of human skill ratings is illustrated in Supplementary Fig. S1.

To assess the extent of consensus between two or more experts that independently rated the same clipping gesture inter-rater reliability was calculated using a one-way random single measure intraclass correlation coefficient (ICC)^27^. Expert skill ratings exhibited an inter-rater reliability of 79% (95% CI 72–85%), a value that is considered excellent^28^.

### Modeling stage 1: instrument detection model

#### Dataset and instrument labeling

101 out of the 949 clip applications from the 242 videos of laparoscopic cholecystectomies were randomly selected. Selected clipping segments were randomly partitioned into a training, a validation and a testing split, with corresponding ratios of 60%, 20% and 20%, respectively. The partitioning was performed based on video segments, i.e., frames from a segment are not distributed across multiple sets.

Frames were extracted from the selected clipping segments at 5 frames per second. The total set was composed of 13,823 individual frames (6950 in training, 3985 in validation and 2888 in testing set). In each frame, grasper and clipper instruments were annotated with a bounding box and a class label. The distribution of frames and object instances are shown in Table 2.

**Table 2:** Distribution of frames and object instances for instrument detection.

	Number of frames	Number of grasper instances	Number of clipper instances
Training	6950	4013	5618
Validation	3985	3027	3351
Testing	2888	2038	2054

#### Model architecture

**Figure 2:**
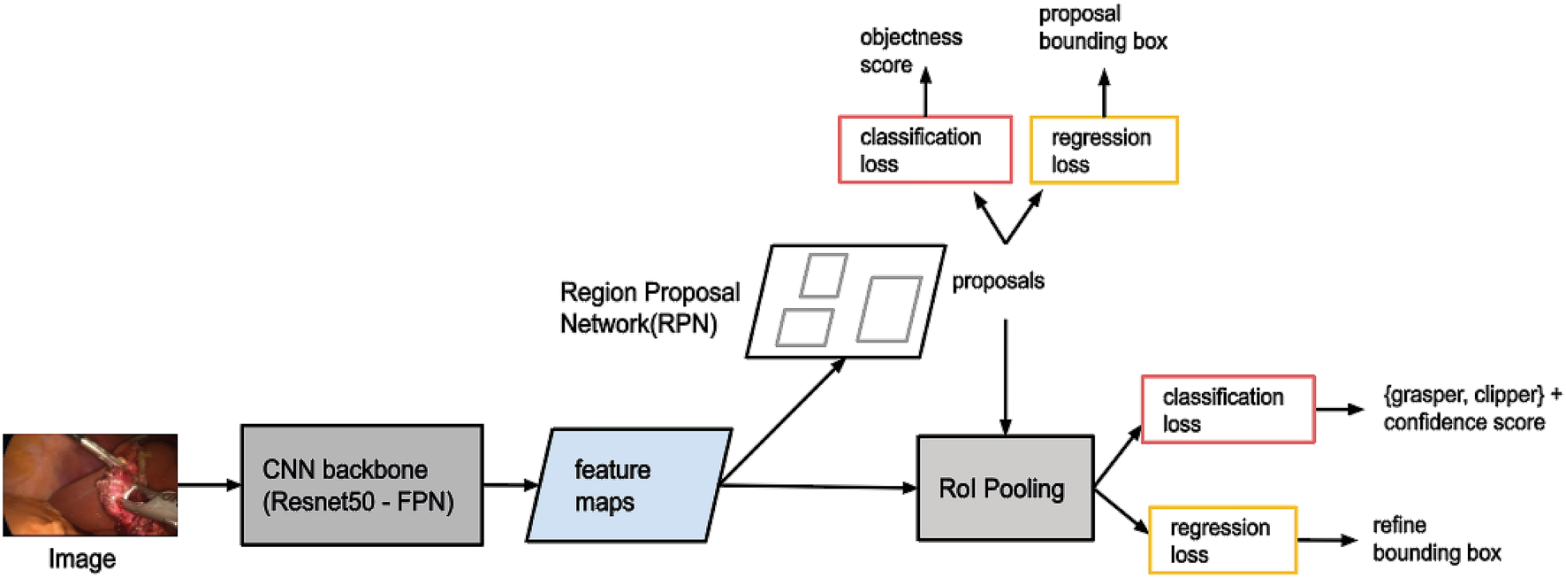
The Feature Pyramid Network (FPN) based Faster R-CNN fine-tuned with surgical instrument locations. The network receives an input of an image of arbitrary size. The backbone network is a Resnet50-FPN CNN which is connected to a Region Proposal Network (RPN) that shares its convolutional layers with the detection network. The RPN is a fully convolutional network which generates region proposals which are highly likely to contain an object. The detection network pools features out of these region proposals and sends them to the final classification and bounding box regression networks. The final output is a bounding box for each detected instrument and a class label (grasper or clipper) with its confidence score.

Recently methods based on deep CNNs have been the top performers in object detection benchmarks^29^. A recent CNN architecture named Feature Pyramid Networks^30^ (FPN) showed top results for generic object detection when combined with Faster R-CNN system^31^, hence, being the basic motivation for our instrument detection model in this work. The original study presented the performance of a 101-layer and 50-layer Resnet (Residual Network) as backbone^30^. We employed the 50-layer Resnet, namely Resnet50-FPN, due to overfitting concerns. The input to the network is an image of arbitrary size. The final output is a bounding box for each detected instrument and a class label (grasper or clipper) with its confidence score. The whole architecture, which is illustrated in Fig. 2, was trained end-to-end.

#### Model training

To initialize the network weights we used transfer learning similar to a previous study^26^. To do so, an instance that had been pre-trained on the 2017 training split of the Microsoft Common Objects in Context (COCO) object detection task (https://cocodataset.org/#detection-2017) was used. The pre-trained model was initially trained on 91 categories. Since we only required two categories (grasper and clipper) the final fully connected classification layer of the pre-trained model was replaced with a new layer that had two outputs and then all layers were retrained.

The network was trained for 15 cycles, using a training batch size of 2. A stochastic gradient descent optimizer was used with an initial learning rate of 0.005, a momentum of 0.9 and a weight decay of 0.0005. Throughout the optimization, the learning rate was halved every 5 cycles. Random horizontal flipping was used to augment our training dataset.

#### Model evaluation

Average precision (AP) and average recall (AR), which have become the standard metrics to evaluate object detection methods^29^, were also used in this work.

To compute AP, predicted bounding boxes are sorted according to their confidence score in descending order. Then, a precision-recall curve is obtained by varying a confidence threshold from 1.0 (highest precision) to 0 (highest recall). AP is computed as the area under the precision-recall curve (AUC). To compute AR, a recall-Intersection over Union (*IoU*) curve is computed by varying an *IoU* threshold between 0.5 (highest recall) and 1.0 (lowest recall) and recall is computed at each level of the threshold. AR is then computed similarly as the area under this curve (AUC).

#### Implementation details

Our implementation is based on the *torchvision* library (https://github.com/pytorch/vision) included in the *PyTorch* framework^32^. We follow best practices from the previous FPN work^30^ to use the same RPN anchor box sizes (5 scales and 3 aspect ratios) and same RPN foreground and background *IoU* thresholds as being 0.7 and 0.3, respectively. Our dataset had videos of two spatial resolutions, i.e., 720 × 576 and 1280 × 720. Before feeding a video frame into the network it was resized such that its shorter side was 800 pixels.

To compute the evaluation metrics, an implementation provided by *torchvision* library was utilized which is based on the evaluation scripts provided by the COCO organization (https://cocodataset.org/#detection-eval). In our evaluation experiments, we both set the detector *IoU* and *confidence* thresholds to 0.5.

### Modeling stage 2: motion feature extraction

#### Preprocessing of instrument locations

The output from the instrument detection model contained the predicted instruments for every frame as well as the x and y coordinates of their associated bounding boxes. This data was initially pre-processed to facilitate the extraction of motion features, as explained in the following.

1. Bounding box coordinates were normalized according to the height and width of the image and the centre location of each bounding box was calculated.
2. Overlapping bounding boxes were removed if the *IoU* of two bounding boxes of the same class was larger than 0.1 or if one of the box areas was smaller than 1.5 times the intersection area of two bounding boxes. These cleaning steps reduced the number of detected instruments per frame and ensured that the same instrument was not detected several times.
3. The particle-tracking library *trackpy* (https://github.com/soft-matter/trackpy)^33^ was used to track the instrument’s location from frame to frame. The most frequently predicted class label of each path was computed, and all instrument detections of the path were assigned to this class. In this way, some of the misclassification from the instrument localization model were cleaned up.
4. Since the focus laid on clipper movements grasper detections were removed. For each frame the clipper detection with the highest confidence was selected as only a single clipper was visible in our videos at any given time.
5. The clipper locations were further smoothed using exponentially moving average.

#### Calculation of motion features

Motion features calculated from the pre-processed instrument locations were aimed to capture the characteristics of good/poor surgical skill. Skilled surgeons are known to handle instruments in a narrow and focused area within their operative field. Poor surgical skill, on the other hand, is indicated by slow, shaky movements with frequent direction changes and larger areas of motion.

To describe the area of motion of the clipper movements the centroid of all clipper locations was calculated as well as the radius from the centroid to all clipper locations throughout the video snippet. The centroid clipper position (with coordinates x and y) is an indication of whether the surgeon’s operative field lies within the centre of the visual field (or image), the radius describes the extent of the movement range of the clipper handling.

To identify whether the surgeon performs directed movements the feature clipper ‘direction change’ was computed which constitutes the percentage of direction changes of at least 45° or more throughout the video snippet. Clipper ‘longest constant direction (LCD)’ refers to the longest consecutive path without direction changes of more than 45°. To further describe the clipper movement magnitude and to identify frequent hesitation clipper ‘position change 1%’ and clipper ‘position change 10%’ were computed which constitute the percentage of clipper location changes of 1 and 10% with respect to the image width/heights.

Additionally, the number of detected clippers per video snippet (clipper count) was computed, a metric correlated to the length of the video snippet, as well as the summed distance of clipper movements throughout the video snippet. A description and visualization of the extracted motion features is given in Supplementary Table S2 and Supplementary Fig. S2.

### Modeling stage 3: skill prediction model

#### Data set and model training

The dataset consisted of ten motion features calculated for each of 949 clipping video segments as well as the associated average skill rating. Prior to training the skill prediction model, five out of the 949 clipping videos were removed due to showing other surgical gestures. Most of clipping segments were rated by more than one expert therefore the average skill rating was calculated.

A linear regression model was trained using the *sklearn* library (https://github.com/scikit-learn/scikit-learn) based on the ten motion features as input and the average skill rating as the dependent variable.

#### Model evaluation

Model performance was assessed using Monte Carlo cross validation with ten random splits of 70% training and 30% testing data.

Two performance metrics were used for evaluation: Accuracy 1/0 and accuracy + 1/− 1. Accuracy 1/0 was used to assess whether the model was able to distinguish good and poor surgical skill. It was calculated by transferring both human skill ratings and automated predictions to binary (a value of 3 or higher from the human expert’s skills rating was considered ‘good’) and computing the percentage of correct cases. The accuracy + 1/− 1 score allowed for a ± 1 deviation from the actual skill rating (e.g. if the human rating is 3 predictions of 4 and 2 are still acceptable).

## Results

To assess surgical skill based on the surgeon’s ability to handle surgical instruments a three-stage modelling approach was developed. The methodology is based on detecting and localizing instruments in surgical videos (stage 1), tracking these instruments over time and calculating relevant motion metrics (stage 2) and predicting surgical skill based on the calculated motion metrics (stage 3). In the following, we will present the results of each of these stages.

As a first step, a frame-wise instrument detection and localization model which predicts the presence, type and location of an instrument in each frame was trained. The model reliably detected clipper and grasper presence and location as exemplified in Fig. 1b (full Video S1 in the Supplementary Information). Detections of the clipper had an average precision (AP) of 78% and an average recall (AR) of 82%. Grasper detections showed even higher AP and AR of 80% and 84% respectively (Differences of AP and AR in validation and test set are listed in Table 3). Further representative examples of challenging situations where the model succeeded (Supplementary Fig. S3) or failed (Supplementary Fig. S4) in detecting and localizing the correct instrument can be found in the Supplementary Information.

**Table 3:** Instrument detection evaluation results.

	Average precision grasper	Average precision clipper	Average recall grasper	Average recall clipper
Validation	0.68	0.86	0.70	0.88
Testing	0.80	0.78	0.84	0.82

As a second step, the outputs from the detection and localization model were first pre-processed before motion metrics, which aimed to capture the characteristics of good/poor surgical skill, were calculated. Pre-processing of the instrument localizations ensured that individual instruments could be tracked throughout the clipping video segment (see “Methods” section for details). The degree of clipper movements varied substantially between video segments (Fig. 1c). Based on the clipper’s movements descriptive motion features like the number of frames the clipper was detected in (clipper count) or the distance the clipper travelled over time (clipper distance) were calculated (see “Methods” section for details). In total, n = 10 motion features were derived.

**Figure 3:**
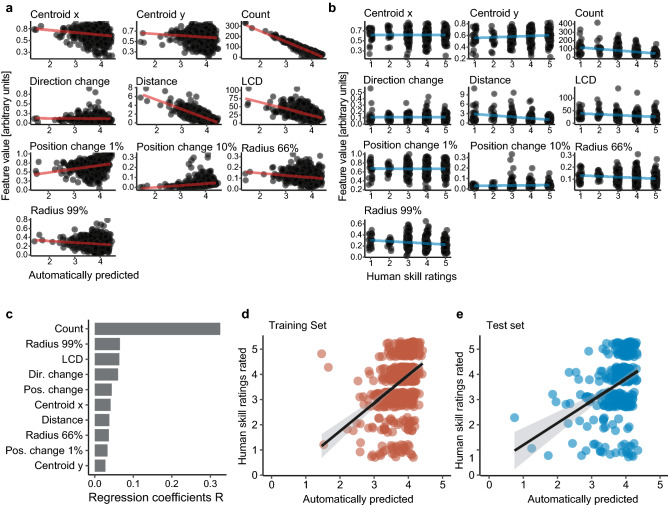
(**a**) Correlations (regression lines in red) of extracted motion features and automatically predicted skill rating in the training set. (**b**) Correlations (regression lines in blue) of extracted motion features and human skill rating in the test set. (**c**) Absolute regression coefficients R of the linear regression model to predict human skill ratings. Correlation of automatically predicted versus human rated skill ratings in the training set (**d**) and test set (**e**).

Some of the clipper motion features showed correlation with human rated skill ratings (Fig. 3a,b, measured using Spearman’s rank correlation coefficient *ρ* with significance level α = 0.05). The motion features ‘Count’ (ρ =  − 0.40 p < 0.001), ‘Distance’ (ρ =  − 0.35 p < 0.001), ‘Radius 66%’ (ρ =  − 0.12 p < 0.001), ‘Radius 99%’ (ρ =  − 0.12 p < 0.001) and ‘Longest constant direction’ (ρ =  − 0.23 p < 0.001) were all negatively correlated with surgical skill ratings. The motion feature ‘Position change 1%’ was positively correlated with surgical skill (ρ = 0.04 p < 0.001). ‘Centroid x’, ‘Centroid y’, ‘Position change 10%’ and ‘Direction change’ showed no significant correlation with the human rated skill ratings.

As the third step, a linear regression model was trained to predict surgical skill based on the extracted motion metrics. The contribution of each feature towards the prediction is shown in Fig. 3c with the ‘clipper count’ being the most important. Predictions of the regression model were evaluated using accuracy 1/0 (binary, good vs. poor surgical skill) and accuracy + 1/− 1 (skill level from 1 to 5, with ± 1 deviation). The linear regression model achieved a performance of 87 ± 0.2% (mean ± SD) in accuracy 1/0 and 70 ± 0.2% in accuracy + 1/− 1. Predictions versus expert rated skill ratings are displayed in Fig. 3d,e. As depicted by the figure, regression line predictions and ground truth labels show a positive correlation.

## Discussion

The presented study aimed to predict surgical skill based on machine learning assisted instrument detection and motion feature extraction of laparoscopic cholecystectomy videos. Since surgical skill is largely determined by smooth and efficient instrument handling our approach is focused on instrument tracking. A three-step modelling approach was performed. An instrument location model was trained that predicts the presence, type and localization of grasper and clipper instruments in a video frame (stage 1). From the clipper localization motion, features that describe the handling of the clipper were derived (stage 2) and a linear regression model was trained to predict surgical skill (stage 3).

In modeling stage 1, the instrument detection and localization model achieved 78% average precision (AP) and 82% average recall (AR) for the clipper on the test set (86% AP and 88% AR for the validation set). Previously published results by Jin et al. reported a higher AP of 86% for clipper identification and bounding box localization in their test set^26^. Visual inspection of Jin et al. dataset suggests that it only contains a single clipper type. Our dataset, in contrast, had two different types of clipper, namely B. Braun Aesculap Challenger and Teleflex Hem-o-lok. Variations in the physical appearance of these two clippers likely made it more challenging for the model to correctly identify clippers thus explaining the lower AP performance compared to Jin et al.

Qualitative results presented in the Supplementary Information (Supplementary Figs. S3, S4) further display that our model performs well in difficult cases such as poor illumination, presence of multiple instruments as well as partial and heavy occlusion. When inspecting incorrect detections, however, it also becomes apparent that difficult instrument angle, very poor illumination or heavy occlusion can prevent the model from correctly identifying and localizing an instrument. To more reliably detect instruments in such difficult situation more examples of occluded and dimly lit instruments will be required as well as specific data augmentation techniques during training.

In modeling stage 2, the calculated motion metrics were compared to expert skill ratings. The number of frames the clipper is present and the distance it travels through the image are negatively correlated with surgical skill rating (Fig. 3b). The motion feature, ‘Count’ is an indicator of duration of clip application. Higher surgical skill rating were associated with a shorter clip application phase. This is not surprising as skilled surgeons spent less time clipping than a less skilled surgeon who has to adjust the clipper position frequently to place the clip correctly. The radius of clipper locations around the centroid is smaller in videos with higher skill ratings (Fig. 3b) demonstrating a narrower movement range of skilled surgeons. Moreover, the largest constant movement direction of the clipper is smaller in higher rated skills (Fig. 3b), indicating that skilled surgeons move their instruments smoothly without tremor or shaky movements.

In modeling stage 3, the accuracy of the machine learning algorithm to predict good or poor surgical skill was 87% and accuracy to predict the skill level ± 1 point was 70%. Of note, even human skill rating considered as gold standard has its limitations in terms of inter-rater reliability with an ICC of 79% in this study.

As shown in Fig. 3e, while there is a correlation between the automated skill ratings and the human rated ground truth values the model fails to predict low and high skill ratings correctly. Low skill was likely difficult to predict as low skill ratings constituted only a small percentage of the dataset (Supplementary Fig. S1), thus making it hard for the model to learn patterns associated with low skill. As the video recordings are from real-life surgery it is comprehensible that low surgical skill ratings are underrepresented in the dataset. A confounding factor for low skill predictions was further that dropping the clip was rated as poor surgical skill (Supplementary Table S1) independent of how well the instrument was handled before the clip was dropped. This poses a problem to our model as it solely relies on instrument movements and has no information on whether the clipper is still loaded with the clip or not.

**Figure 4:**
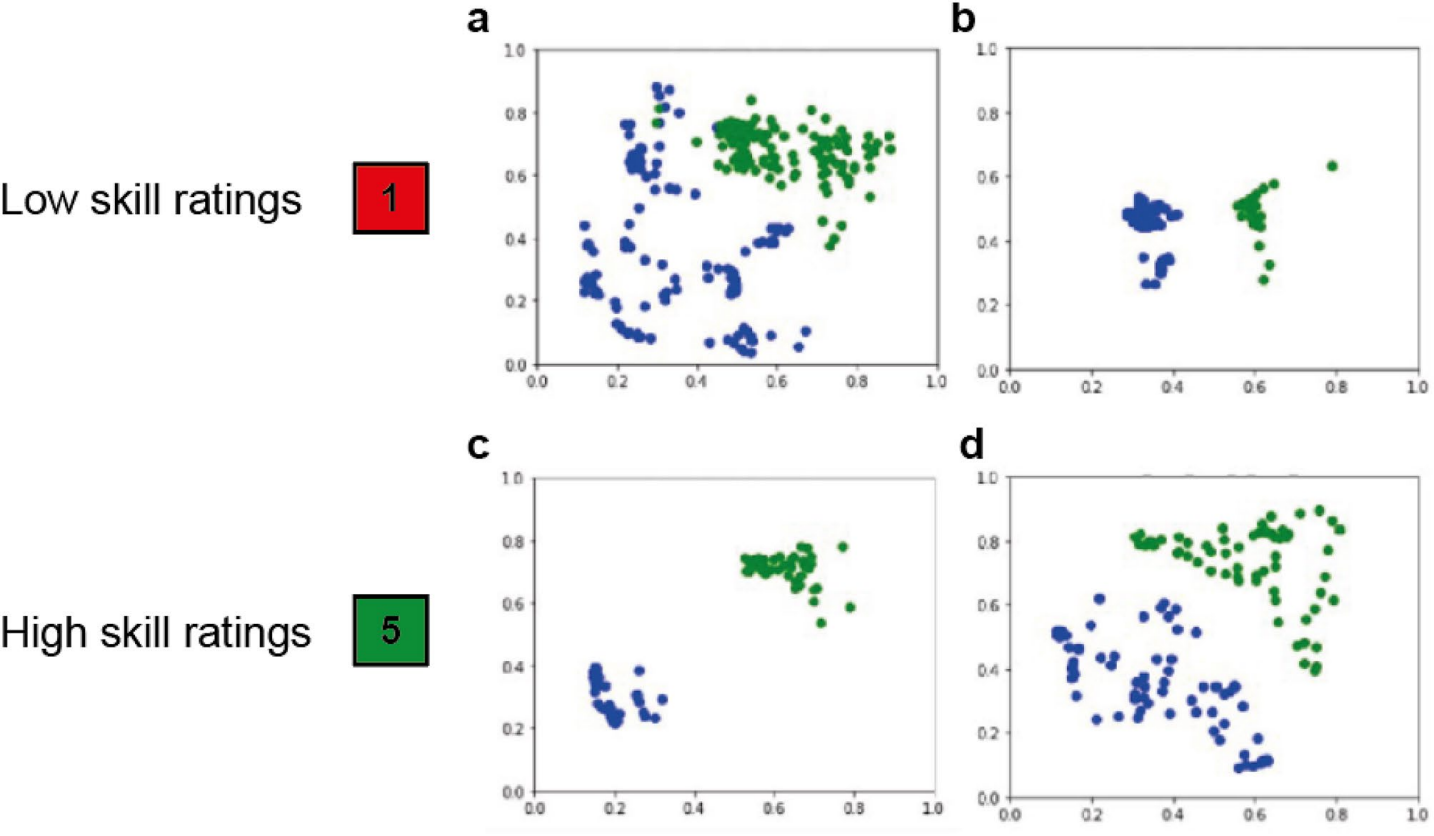
Examples on how camera movement and zoom affect instrument localizations (blue: grasper, green: clipper). (**a**) Low surgical skill rating and dispersed movement pattern. (**b**) Low surgical skill rating and precise movement pattern (clip lost). (**c**) High skill rating and precise movement pattern (camera zoomed out). (**d**) High skill rating and dispersed movement pattern (camera zoomed in).

When looking at instrument localization plots it further becomes apparent that the calculated features are strongly affected by camera movement and zoom. Figure 4 shows examples of instrument locations for low (Fig. 4a,b) and high skill ratings (Fig. 4c,d). In example a the localizations are dispersed, the clipper and grasper both have large movement range suggesting that the surgeon had problems finding the best position to apply the clip, thus justifying a low rating. Example b, an example of high skill, on the other hand shows a narrow movement range indicating clean instrument handling while the video received a low skill rating due to the clip being lost. Similarly, example c and d show quite different movement ranges suggesting different skill levels. However, in example c the camera was zoomed out so that the instrument movement appeared small while the camera was zoomed in further in example d, thus wrongly indicating a large movement range. To improve model performance and render the calculated features more meaningful camera movement needs to be stabilized and zoom factor corrected.

Additionally, while instrument handling is an important factor to assess surgical skill, tissue handling and difficulty of the operation also influences surgical skill level, which is not considered in the current work.

## Conclusion

Automated surgical skill assessment using the proposed three stage machine learning algorithm is effective to distinguish good and poor surgical skill with an accuracy of 87 ± 0.2%. The current algorithm, however, has limitations to predict the exact surgical skill level. Therefore, a larger training database and refinement of algorithm is required to further improve automated surgical skill assessment.

## Supplementary Information

Supplementary Information 1.

Supplementary Video 1.

## Data Availability

The data that support the findings of this study are under a non-published license and are not publicly available.
