# Author Correction: Automation of surgical skill assessment using a three-stage machine learning algorithm

**DOI:** 10.1038/s41598-021-88175-x

**Published:** 2021-04-20

**Authors:** Joël L. Lavanchy, Joel Zindel, Kadir Kirtac, Isabell Twick, Enes Hosgor, Daniel Candinas, Guido Beldi

**Affiliations:** 1 Department of Visceral Surgery and Medicine, Inselspital, Bern University Hospital, University of Bern, 3010 Bern, Switzerland; 2 Caresyntax, Komturstr. 18A, 12099 Berlin, Germany

Correction to: *Scientific Reports* https://doi.org/10.1038/s41598-021-84295-6, published online 04 March 2021

The original version of this Article contained errors in the Reference list. The authors omitted the below paper, which is listed as Reference 22.

Sarikaya, D., Corso, J. J. & Guru, K. A. Detection and localization of robotic tools in robot-assisted surgery videos using deep neural networks for region proposal and detection. *IEEE Trans. Med. Imaging* **36**, 1542–1549 10.1109/TMI.2017.2665671 (2017).

As a result, in the Introduction,

“Methodologies have ranged from hidden markov chains^20^ and traditional machine learning classifiers^14^, over time series feature extraction^17,18^ to CNNs^15,16,21^.^”^

now reads:

“Methodologies have ranged from hidden markov chains^20^ and traditional machine learning classifiers^14^, over time series feature extraction^17,18^ to CNNs^15,16,21,22^.”

Consequently, References 23–33 were incorrectly listed as References 22–32.

The original Article has been corrected.

