# Supplementary material for: Automation of surgical skill assessment using a three-stage machine learning algorithm
Source: Sci Rep. 2021 Mar 4;11:5197. doi: 10.1038/s41598-021-84295-6 (PMC7933408; doi:10.1038/s41598-021-84295-6)
Supplement: Supplementary file 1 — Supplementary Information 1. [file 41598_2021_84295_MOESM1_ESM.pdf]

Automation of Surgical Skill Assessment Using a Three-Stage Machine Learning Algorithm

Joël L. Lavanchy[1], Joel Zindel[1]; Kadir Kirtac[2]; Isabell Twick[2]; Enes Hosgor[2]; Daniel Candinas[1]; Guido Beldi[1]

1 Department of Visceral Surgery and Medicine, Inselspital, Bern University Hospital, University of Bern, CH-3010 Bern, Switzerland

2 Caresyntax, Komturstr. 18A, D-12099 Berlin, Germany

Supplementary Information

Table S1. Definitions of skill rating

Rating	Definition
5	Clip was applied in the right position in a straight manner and the tip of the clip was visualized before closure
4	Clip was applied in the right position, movement was steady but slow
3	Clip was applied in the right position, but movement was hesitating and/or jittering
2	Clip was applied in the wrong position and/or with shaky movements
1	Clip was lost or not applied at all

Table S2. Definition of the extracted clipper motion features

Feature name	Explanation
Count	Number of frames the clipper was detected in the video snippet
Distance	Distance travelled by the clipper throughout the video snippet
Centroid x,<br>Centroid y	X and y coordinates of the location centroid (= the centre of all the clipper locations throughout the video snippet)
Radius 66%,<br>Radius 99%	Clipper movement radius around its centroid. (Radius 66%/99% refers to the radius from the centroid for the 66%/99% closest location points, respectively)
Direction change	Percentage of clipper direction changes of 45° or more throughout the video snippet
Longest constant direction (LCD)	Longest consecutive clipper path without direction changes of more than 45°
Position change 1%,<br>Position change 10%	Percentage of clipper location changes of 1 and 10% with respect to the image width/heights

Figure S1. a Illustration of the Likert scale used to rate surgical skills: The minimum score 1 indicates poor surgical skill, the maximum score indicates 5 excellent surgical skill. **b** Distribution of human skills ratings in the dataset.

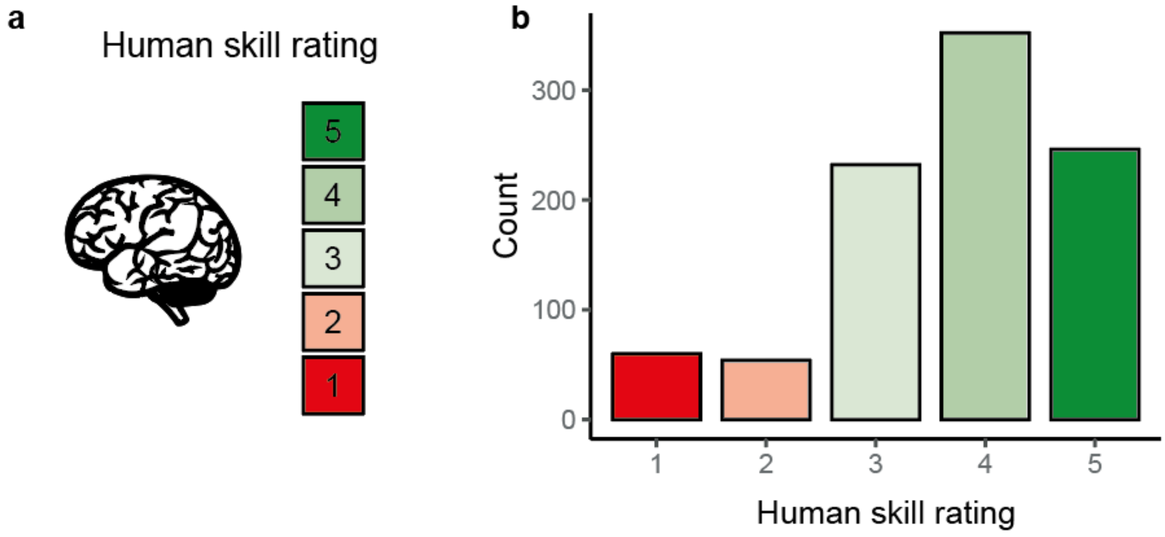

Figure S2. Visualization of the extracted clipper motion features

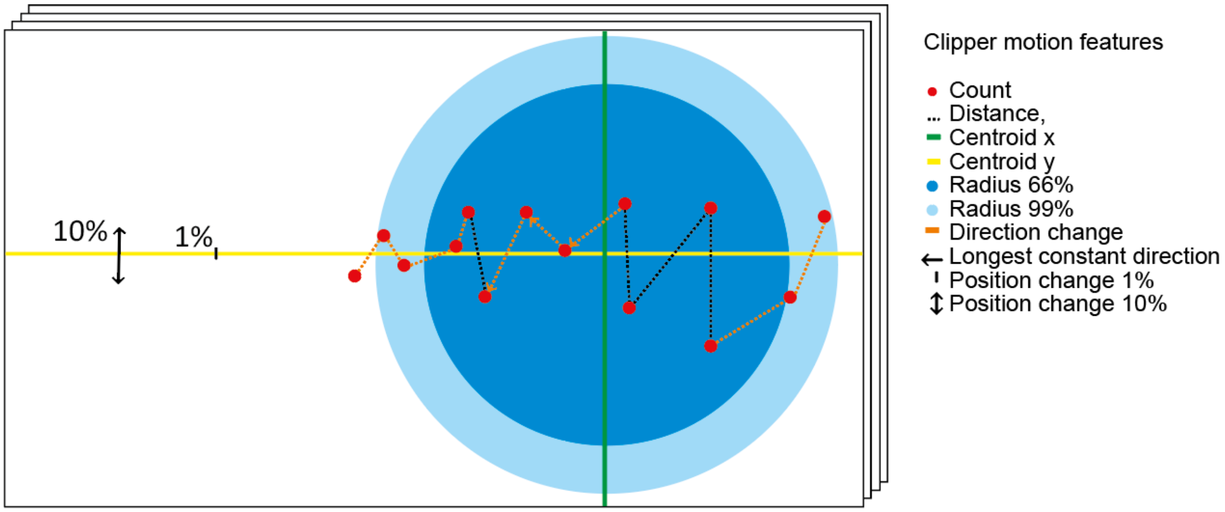

Figure S3. Example frames where the instrument detection model succeeded. True positive grasper identification **a** despite heavy smoke **b** and despite other instrument (scissor) visible. True positive clipper identification **c** despite partial occlusion of the clipper, **d** despite full occlusion of the clipper, **e** despite bad bad angle **f** and despite difficult angle and blur.

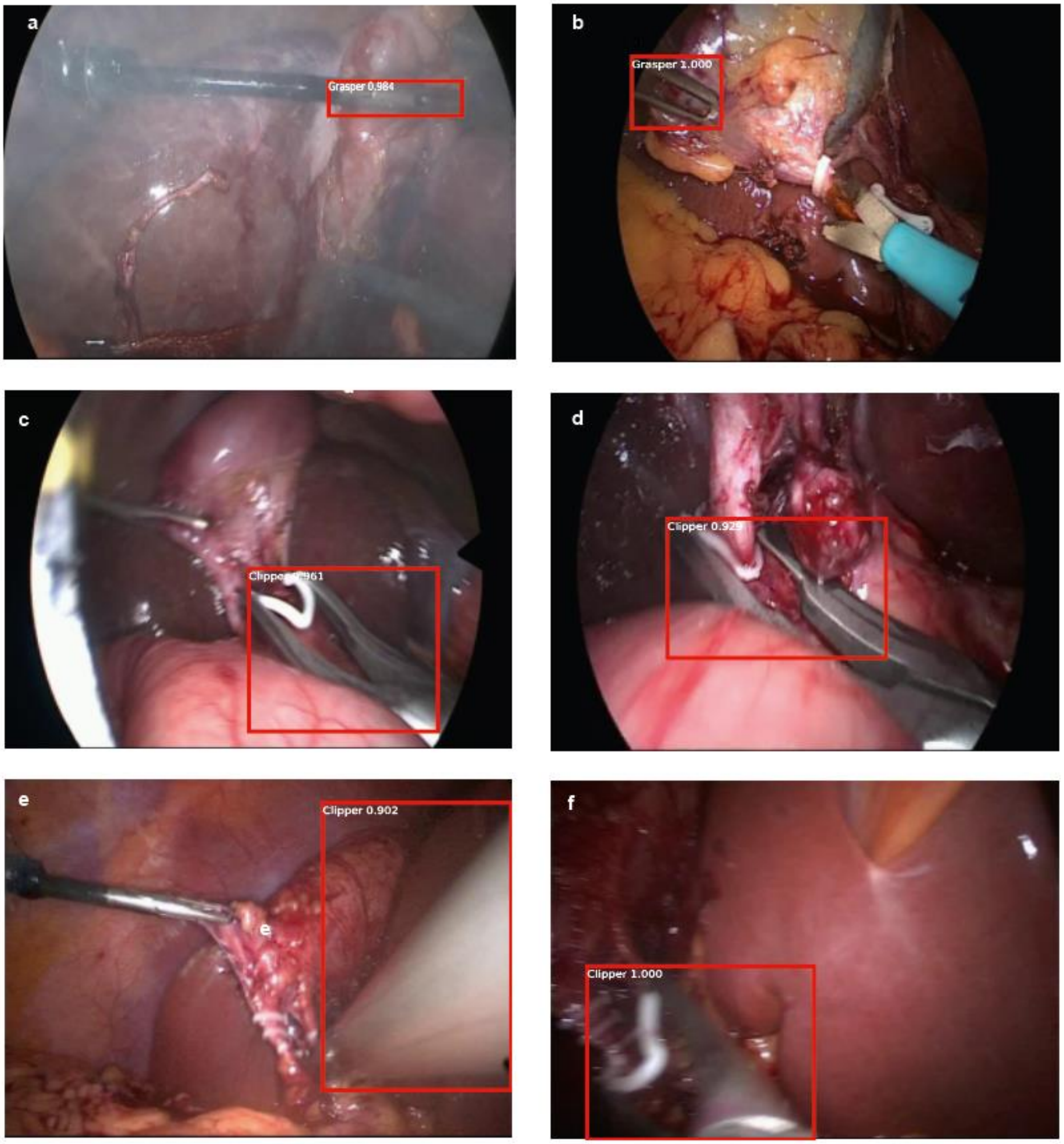

Figure S4. Example frames where the instrument detection model failed. **a** False negative clipper detection due to bad angle and blur **b** False negative grasper detection due to multiple visible graspers **c** False positive clipper identification **d** False negative B.Braun Aesculap clipper detection **e** False negative Teleflex Hem-o-lok clipper detection **f** False negative clipper identification due to clipper occlusion.

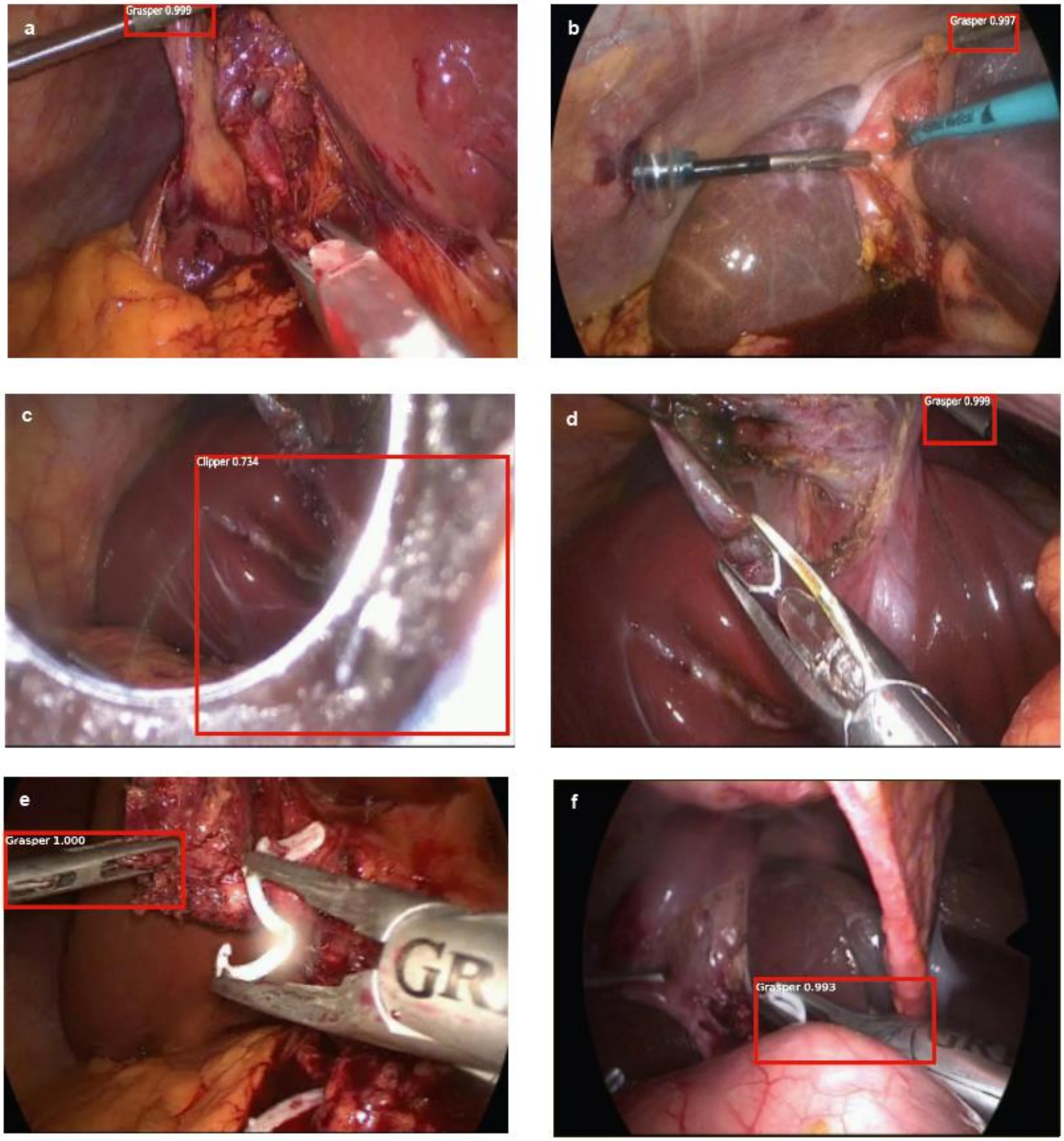

Video S1. Example video snippet with instrument detection algorithm. Instruments are labeled with predicted bounding boxes, class labels and prediction accuracies

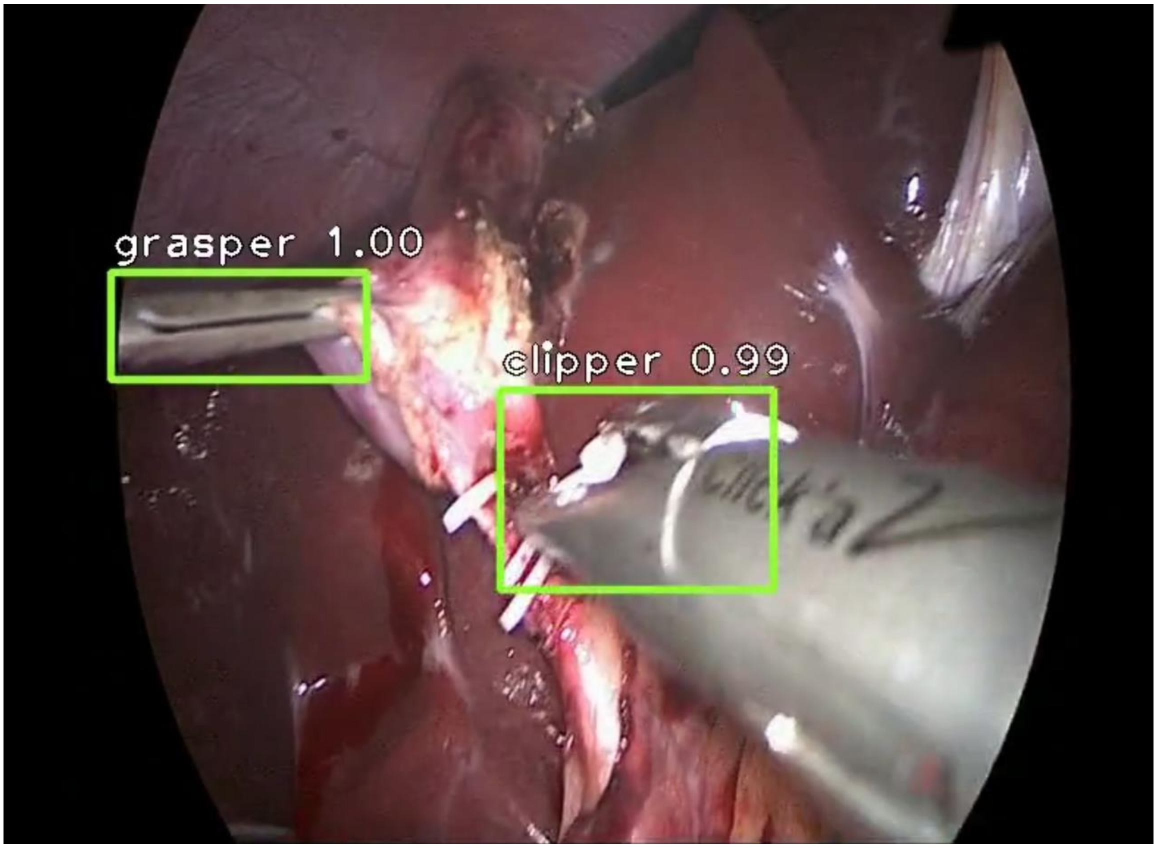

Filename: *motion_tracking_25fps.mp4*
